# Supplementary material for: Impact of crisis intervention on mental health in the context of specific civilian emergencies
Source: PLoS One. 2025 Sep 10;20(9):e0331249. doi: 10.1371/journal.pone.0331249 (PMC12422494; doi:10.1371/journal.pone.0331249)
Supplement: S2 File — (DOCX) [file pone.0331249.s002.docx]

**Supplementary 2**. Selected documents for in-depth analysis

1. Teller JLS, Munetz MR, Gil KM, Ritter C. Crisis intervention team training for police officers responding to mental disturbance calls. Psychiatr Serv. 2006;57: 232–237. doi:10.1176/appi.ps.57.2.232

2. Comartin EB, Swanson L, Kubiak S. Mental Health Crisis Location and Police Transportation Decisions: The Impact of Crisis Intervention Team Training on Crisis Center Utilization. J Contemp Crim Justice. 2019;35: 241–260. doi:10.1177/1043986219836595

3. Kubiak S, Shamrova D, Comartin E. Enhancing knowledge of adolescent mental health among law enforcement: Implementing youth-focused crisis intervention team training. Eval Program Plann. 2019;73: 44–52. doi:10.1016/j.evalprogplan.2018.11.006

4. Demir B, Broussard B, Goulding SM, Compton MT. Beliefs about causes of schizophrenia among police officers before and after crisis intervention team training. Community Ment Health J. 2009;45: 385–392. doi:10.1007/s10597-009-9194-7

5. Mcneeley S, Donley C. Crisis Intervention Team Training in a Correctional Setting: Examining Compliance, Mental Health Referrals, and Use of Force. Crim Justice Behav. 2021;48: 195–214. doi:10.1177/0093854820959394

6. Ahn E, Kim J, Moon S, Ko YH, Cho H, Park JH, et al. Effect of a Crisis Intervention Team for suicide attempt patients in an emergency department in Korea. Hong Kong J Emerg Med. 2020;27: 92–98. doi:10.1177/1024907918822255

7. Boazak M, Yoss S, Kohrt BA, Gwaikolo W, Strode P, Compton MT, et al. Law enforcement and mental health clinician partnerships in global mental health: outcomes for the Crisis Intervention Team (CIT) model adaptation in Liberia, West Africa. Glob Ment Heal. 2020;7: 41–44. doi:10.1017/gmh.2019.31

8. Martin-Iñigo L, Ortiz S, Urbano D, Teba Pérez S, Contaldo SF, Alvarós J, et al. Assessment of the efficacy of a Crisis Intervention Team (CIT): experience in the Esplugues Mental Health Center (Barcelona). Soc Psychiatry Psychiatr Epidemiol. 2022;57: 2109–2117. doi:10.1007/s00127-022-02250-w

9. Newton H, Beetham T, Busch SH. Association of Access to Crisis Intervention Teams With County Sociodemographic Characteristics and State Medicaid Policies and Its Implications for a New Mental Health Crisis Lifeline. JAMA Netw Open. 2022;5: E2224803. doi:10.1001/jamanetworkopen.2022.24803

10. Nick GA, Williams S, Lekas H-M, Pahl K, Blau C, Kamin D, et al. Crisis Intervention Team (CIT) training and impact on mental illness and substance use-related stigma among law enforcement. Drug Alcohol Depend Reports. 2022;5: 100099. doi:10.1016/j.dadr.2022.100099

11. Koziarski J, O’Connor C, Frederick T. Policing mental health: The composition and perceived challenges of Co-response Teams and Crisis Intervention Teams in the Canadian context. Police Pract Res. 2020; 1–19. doi:10.1080/15614263.2020.1786689

12. Azami G, Mozafari A, Kafashian M, Aazami S, Ebrahimy B. Helping a Patient With a Pre-Existing Mental Health Condition Cope With Depression and COVID-19 Using the Neuman Systems Model: A Single Intrinsic Case Study. Creat Nurs. 2023;29: 295–302. doi:10.1177/10784535231211694

13. Foo CYS, Verdeli H, Tay AK. Psychosocial interventions for occupational stress and psychological disorders in humanitarian aid and disaster responders: A critical review. Handbook of Cognitive Behavioral Therapy by Disorder: Case Studies and Application for Adults. Elsevier Inc.; 2023. doi:10.1016/B978-0-323-85726-0.00008-9

14. Dekel I, Hertz-Palmor N, Dorman-Ilan S, Reich-Dvori M, Gothelf D, Pessach IM. Bridging the gap between the emergency department and outpatient care: feasibility of a short-term psychiatric crisis intervention for children and adolescents. Eur Child Adolesc Psychiatry. 2023;32: 631–637. doi:10.1007/s00787-021-01896-2

15. He C, Chang S, Lu Y, Zhang H, Zhou H, Guo Y, et al. Effects of Online Psychological Crisis Intervention for Frontline Nurses in COVID-19 Pandemic. Front Psychiatry. 2022;13: 1–6. doi:10.3389/fpsyt.2022.937573

16. Zhang N, Shi W, Feng D, Fang W, Zeng Q, Qu Y. A preliminary study on the anxiety and depression situation and psychological intervention of the first-line medical staff in our hospital during the COVID-19 epidemic. J Clin Neurosci. 2021;91: 9–12. doi:10.1016/j.jocn.2021.06.037

17. Zhong B, Huang Y, Liu Q. Mental health toll from the coronavirus: Social media usage reveals Wuhan residents’ depression and secondary trauma in the COVID-19 outbreak. Comput Human Behav. 2021;114: 106524. doi:10.1016/j.chb.2020.106524

18. Miniguano-Trujillo A, Salazar F, Torres R, Arias P, Sotomayor K. An integer programming model to assign patients based on mental health impact for tele-psychotherapy intervention during the Covid–19 emergency. Health Care Manag Sci. 2021;24: 286–304. doi:10.1007/s10729-020-09543-z

19. Schweizer S, Lawson RP, Blakemore SJ. Uncertainty as a driver of the youth mental health crisis. Curr Opin Psychol. 2023;53: 101657. doi:10.1016/j.copsyc.2023.101657

20. Schulte C, Sextl-Plötz T, Baumeister H, Titzler I, Sander LB, Sachser C, et al. What to do when the unwanted happens? Negative event management in studies on internet- and mobile-based interventions for youths and adults with two case reports. Internet Interv. 2024;35. doi:10.1016/j.invent.2024.100710

21. Ruggeri M, Salvi G, Perwanger V, Phelan M, Pellegrini N, Parabiaghi A. Satisfaction with community and hospital-based emergency services amongst severely mentally ill service users: A comparison study in South-Verona and South-London. Soc Psychiatry Psychiatr Epidemiol. 2006;41: 302–309. doi:10.1007/s00127-006-0030-x

22. Adams K, Shakespeare-Finch J, Armstrong D. An Interpretative Phenomenological Analysis of Stress and Well-Being in Emergency Medical Dispatchers. J Loss Trauma. 2015;20: 430–448. doi:10.1080/15325024.2014.949141

23. North CS, Pfefferbaum B. Mental health response to community disasters: A systematic review. Jama. 2013;310: 507–518. doi:10.1001/jama.2013.107799

24. Humer E, Pieh C, Probst T, Kisler IM, Schimböck W, Schadenhofer P. Telephone emergency service 142 (Telefonseelsorge) during the covid-19 pandemic–cross-sectional survey among counselors in austria. Int J Environ Res Public Health. 2021;18: 1–10. doi:10.3390/ijerph18052228

25. Haas LJ, Benedict JG, Kobos JC. Psychotherapy by telephone: Risks and benefits for psychologists and consumers. Prof Psychol Res Pract. 1996;27: 154–160. doi:10.1037/0735-7028.27.2.154

26. Ribeiro E, Sampaio A, Gonçalves MM, Taveira MDC, Cunha J, Maia Â, et al. Telephone-based psychological crisis intervention: the Portuguese experience with COVID-19. Couns Psychol Q. 2021;34: 432–446. doi:10.1080/09515070.2020.1772200

27. Wang W, Chen DD, Yang Y, Liu X, Miao D. A study of psychological crisis intervention with family members of patients who died after emergency admission to hospital. Soc Behav Pers. 2010;38: 469–478. doi:10.2224/sbp.2010.38.4.469

28. Yang H, Ma J, Hu H, Li F. Identification, trend analysis and influencing factors of mental health status of the Chinese older adults. Int J Environ Res Public Health. 2020;17: 1–14. doi:10.3390/ijerph17218251

29. Xiao R, Zhang C, Lai Q, Hou Y, Zhang X. Applicability of the Dual-Factor Model of Mental Health in the Mental Health Screening of Chinese College Students. Front Psychol. 2021;11: 1–7. doi:10.3389/fpsyg.2020.549036

30. Jiang Y, Ding C, Shen B. Latent Profile Analysis of Mental Health among Chinese University Students: Evidence for the Dual-Factor Model. Healthc. 2023;11. doi:10.3390/healthcare11202719

31. Boscarino JA, Adams RE, Figley CR. A prospective cohort study of the effectiveness of employer-sponsored crisis interventions after a major disaster. Int J Emerg Ment Health. 2005;7: 9–22.

32. Wesemann U, Mahnke M, Polk S, Bühler A, Willmund G. Impact of Crisis Intervention on the Mental Health Status of Emergency Responders Following the Berlin Terrorist Attack in 2016. Disaster Med Public Health Prep. 2019;14: 168–172. doi:10.1017/dmp.2019.60

33. Battles ED. An Exploration of Post-traumatic Stress Disorder in Emergency Nurses Following Hurricane Katrina. J Emerg Nurs. 2007;33: 314–318. doi:10.1016/j.jen.2007.01.008

34. Leon SL, Cappelli M, Ali S, Craig W, Curran J, Gokiert R, et al. The current state of mental health services in Canada’s paediatric emergency departments. Paediatr Child Heal. 2013;18: 81–85. doi:10.1093/pch/18.2.81

35. Wambua GN, Falkenström F, Kumar M, Cuijpers P. Outcome evaluation of psychological interventions offered to adolescents seeking mental health services at the national referral and teaching hospital in Nairobi, Kenya. SSM - Ment Heal. 2022;2. doi:10.1016/j.ssmmh.2022.100137

36. Farran N. Mental health in Lebanon: Tomorrow’s silent epidemic. Ment Heal Prev. 2021;24: 200218. doi:10.1016/j.mhp.2021.200218

37. Simpson S. A Single-session Crisis Intervention Therapy Model for Emergency Psychiatry. Clin Pract Cases Emerg Med. 2019;3: 27–32. doi:10.5811/cpcem.2018.10.40443

38. Weiss J, Lunsky Y. Service utilization patterns in parents of youth and adults with intellectual disability who experienced behavioral crisis. J Ment Health Res Intellect Disabil. 2010;3: 145–163. doi:10.1080/19315864.2010.490617

39. Yibeltal Yedemie Y. Mental Health and Psychosocial Aspects of Corona Virus Disease (COVID-19) Outbreak in Ethiopia: Psychological Intervention for Public Psychological Crisis. Int J Psychol Brain Sci. 2020;5: 56. doi:10.11648/j.ijpbs.20200504.11

40. Miller AB, Oppenheimer CW, Chew RF, Weitzel KJ, D’Arcangelo B, Barnes A, et al. Exploring whether mental health crisis text conversations that include discussion of firearms differ from those without firearms. Prev Med (Baltim). 2023;177: 107783. doi:10.1016/j.ypmed.2023.107783

41. Mukhtar S. Mental Health and Psychosocial Aspects of Coronavirus Outbreak in Pakistan: Psychological Intervention for Public Mental Health Crisis. Asian J Psychiatr. 2020;51: 102069. doi:10.1016/j.ajp.2020.102069

42. Senneseth M, Alsaker K, Natvig GK. Health-related quality of life and post-traumatic stress disorder symptoms in accident and emergency attenders suffering from psychosocial crises: A longitudinal study. J Adv Nurs. 2012;68: 402–413. doi:10.1111/j.1365-2648.2011.05752.x

43. Clary B, Baert B, Bourrel G, Amouyal M, Lognos B, Oude-Engberink A, et al. Integrating general practitioners into crisis management would accelerate the transition from victim to effective professional: Qualitative analyses of a terrorist attack and catastrophic flooding. Eur J Gen Pract. 2022;28: 125–133. doi:10.1080/13814788.2022.2072826

44. Cloutier P, Thibedeau N, Barrowman N, Gray C, Kennedy A, Leon SL, et al. Predictors of repeated visits to a pediatric emergency department crisis intervention program. Can J Emerg Med. 2017;19: 122–130. doi:10.1017/cem.2016.357

45. Park JM, Bae SM. Impact of depressive, anxiety, and PTSD symptoms in disaster victims on quality of life: The moderating effect of perceived community resilience. Int J Disaster Risk Reduct. 2022;69: 102749. doi:10.1016/j.ijdrr.2021.102749

46. Wilson JP, Raphael B, Meldrum L, Bedosky C, Sigman M. Preventing PTSD in trauma survivors. Bull Menninger Clin. 2000;64: 181–196.

47. Lewis SJ. Do one-shot preventive interventions for PTSD work? A systematic research synthesis of psychological debriefings. Aggress Violent Behav. 2003;8: 329–343. doi:10.1016/S1359-1789(01)00079-9

48. Chatzea VE, Sifaki-Pistolla D, Vlachaki SA, Melidoniotis E, Pistolla G. PTSD, burnout and well-being among rescue workers: Seeking to understand the impact of the European refugee crisis on rescuers. Psychiatry Res. 2018;262: 446–451. doi:10.1016/j.psychres.2017.09.022

49. Carmassi C, Foghi C, Dell’Oste V, Cordone A, Bertelloni CA, Bui E, et al. PTSD symptoms in healthcare workers facing the three coronavirus outbreaks: What can we expect after the COVID-19 pandemic. Psychiatry Res. 2020;292: 113312. doi:10.1016/j.psychres.2020.113312

50. Megalakaki O, Kokou-Kpolou CK, Vaudé J, Park S, Iorfa SK, Cénat JM, et al. Does peritraumatic distress predict PTSD, depression and anxiety symptoms during and after COVID-19 lockdown in France? A prospective longitudinal study. J Psychiatr Res. 2021;137: 81–88. doi:10.1016/j.jpsychires.2021.02.035

51. Tang W, Hu T, Hu B, Jin C, Wang G, Xie C, et al. Prevalence and correlates of PTSD and depressive symptoms one month after the outbreak of the COVID-19 epidemic in a sample of home-quarantined Chinese university students. J Affect Disord. 2020;274: 1–7. doi:10.1016/j.jad.2020.05.009

52. Qian J, Zhou X, Sun X, Wu M, Sun S, Yu X. Effects of expressive writing intervention for women’s PTSD, depression, anxiety and stress related to pregnancy: A meta-analysis of randomized controlled trials. Psychiatry Res. 2020;288: 112933. doi:10.1016/j.psychres.2020.112933

53. Maalouf FT, Haidar R, Mansour F, Elbejjani M, Khoury J El, Khoury B, et al. Anxiety, depression and PTSD in children and adolescents following the Beirut port explosion. J Affect Disord. 2022;302: 58–65. doi:10.1016/j.jad.2022.01.086

54. Figueroa RA, Cortés PF, Marín H, Vergés A, Gillibrand R, Repetto P. The ABCDE psychological first aid intervention decreases early PTSD symptoms but does not prevent it: results of a randomized-controlled trial. Eur J Psychotraumatol. 2022;13. doi:10.1080/20008198.2022.2031829

55. Wu Y, Dai Z, Jing S, Liu X, Zhang L, Liu X, et al. Prevalence and influencing factors of PTSD symptoms among healthcare workers: A multicenter cross-sectional study during the surge period of the COVID-19 pandemic since December 2022 in the Chinese mainland. J Affect Disord. 2024;348: 70–77. doi:10.1016/j.jad.2023.12.008

56. Djatche JM, Herrington OD, Nzebou D, Galusha D, Boum Y, Hassan S. A cross-sectional analysis of mental health disorders in a mental health services-seeking population of children, adolescents, and young adults in the context of ongoing violence and displacement in northern Cameroon. Compr Psychiatry. 2022;113. doi:10.1016/j.comppsych.2021.152293

57. Sotoudeh HG, Alavi SS, Akbari Z, Jannatifard F, Artounian V. The effect of brief crisis intervention package on improving quality of life and mental health in patients with COVID-19. Iran J Psychiatry. 2020;15: 205–212. doi:10.18502/ijps.v15i3.3812

58. Singh L, Kanstrup M, Gamble B, Geranmayeh A, Göransson KE, Rudman A, et al. A first remotely-delivered guided brief intervention to reduce intrusive memories of psychological trauma for healthcare staff working during the ongoing COVID-19 pandemic: Study protocol for a randomised controlled trial. Contemp Clin Trials Commun. 2022;26. doi:10.1016/j.conctc.2022.100884

59. Pappa S, Sakkas N, Sakka E. A year in review: sleep dysfunction and psychological distress in healthcare workers during the COVID-19 pandemic. Sleep Med. 2022;91: 237–245. doi:10.1016/j.sleep.2021.07.009

60. Cénat JM, Farahi SMMM, Dalexis RD, Darius WP, Bekarkhanechi FM, Poisson H, et al. The global evolution of mental health problems during the COVID-19 pandemic: A systematic review and meta-analysis of longitudinal studies. J Affect Disord. 2022;315: 70–95. doi:10.1016/j.jad.2022.07.011

61. Eweida RS, Rashwan ZI, Khonji LM, Shalhoub AA Bin, Ibrahim N. Psychological first aid intervention: rescue from psychological distress and improving the pre-licensure nursing students’ resilience amidst COVID-19 crisis and beyond. Sci African. 2023;19. doi:10.1016/j.sciaf.2022.e01472

62. Stapleton AB, Lating J, Kirkhart M, Everly GS. Effects of medical crisis intervention on anxiety, depression, and posttraumatic stress symptoms: A meta-analysis. Psychiatr Q. 2006;77: 231–238. doi:10.1007/s11126-006-9010-2

63. Giummarra MJ, Lennox A, Dali G, Costa B, Gabbe BJ. Early psychological interventions for posttraumatic stress, depression and anxiety after traumatic injury: A systematic review and meta-analysis. Clin Psychol Rev. 2018;62: 11–36. doi:10.1016/j.cpr.2018.05.001

64. Marvaldi M, Mallet J, Dubertret C, Moro MR, Guessoum SB. Anxiety, depression, trauma-related, and sleep disorders among healthcare workers during the COVID-19 pandemic: A systematic review and meta-analysis. Neurosci Biobehav Rev. 2021;126: 252–264. doi:10.1016/j.neubiorev.2021.03.024

65. Zheng R, Zhou Y, Fu Y, Xiang Q, Cheng F, Chen H, et al. Prevalence and associated factors of depression and anxiety among nurses during the outbreak of COVID-19 in China: A cross-sectional study. Int J Nurs Stud. 2021;114: 103809. doi:10.1016/j.ijnurstu.2020.103809

66. Chen X, Liu P, Lei GF, Tong L, Wang H, Zhang XQ. Sleep quality and the depression-anxiety-stress state of frontline nurses who perform nucleic acid sample collection during COVID-19: A cross-sectional study. Psychol Res Behav Manag. 2021;14: 1889–1900. doi:10.2147/PRBM.S338495

67. Mahmud S, Hossain S, Muyeed A, Islam MM, Mohsin M. The global prevalence of depression, anxiety, stress, and, insomnia and its changes among health professionals during COVID-19 pandemic: A rapid systematic review and meta-analysis. Heliyon. 2021;7: e07393. doi:10.1016/j.heliyon.2021.e07393

68. Wechsler TF, Schmidmeier M, Biehl S, Gerczuk J, Guerrero-Cerda FM, Mühlberger A. Individual changes in stress, depression, anxiety, pathological worry, posttraumatic stress, and health anxiety from before to during the COVID-19 pandemic in adults from Southeastern Germany. BMC Psychiatry. 2022;22: 1–20. doi:10.1186/s12888-022-04148-y

69. Ibrahim UU, Abubakar Aliyu A, Abdulhakeem OA, Abdulaziz M, Asiya M, Sabitu K, et al. Prevalence of Boko Haram crisis related depression and post-traumatic stress disorder symptomatology among internally displaced persons in Yobe state, North East, Nigeria. J Affect Disord Reports. 2023;13. doi:10.1016/j.jadr.2023.100590

70. Sun JL. Construction of Mental Health Education and Psychological Crisis Intervention System in Higher Vocational Colleges. Psychiatr Danub. 2021;33: S170–S172.

71. Boscarino JA. Community Disasters, Psychological Trauma, and Crisis Intervention. Int J Emerg Ment Health. 2015;17: 369–371.

72. Koranda NW, Knettel BA, Mabula P, Joshi R, Kisigo G, Klein C, et al. Evaluating the impact of a training program in prehospital trauma care and mental health for traffic police in Arusha, Tanzania. Int Emerg Nurs. 2023;70: 101346. doi:10.1016/j.ienj.2023.101346

73. Fan Y, Shi Y, Zhang J, Sun D, Wang X, Fu G, et al. The effects of narrative exposure therapy on COVID-19 patients with post-traumatic stress symptoms: A randomized controlled trial. J Affect Disord. 2021;293: 141–147. doi:10.1016/j.jad.2021.06.019

74. Nuryana Z, Xu W, Kurniawan L, Sutanti N, Makruf SA, Nurcahyati I. Student stress and mental health during online learning: Potential for post-COVID-19 school curriculum development. Compr Psychoneuroendocrinology. 2023;14: 100184. doi:10.1016/j.cpnec.2023.100184

75. Pigeon WR, Heffner KL, Crean H, Gallegos AM, Walsh P, Seehuus M, et al. Responding to the need for sleep among survivors of interpersonal violence: A randomized controlled trial of a cognitive-behavioral insomnia intervention followed by PTSD treatment. Contemp Clin Trials. 2015;45: 252–260. doi:10.1016/j.cct.2015.08.019

76. Werner EA, Aloisio CE, Butler AD, D’Antonio KM, Kenny JM, Mitchell A, et al. Addressing mental health in patients and providers during the COVID-19 pandemic. Semin Perinatol. 2020;44: 151279. doi:10.1016/j.semperi.2020.151279

77. Singewald N, Sartori SB, Reif A, Holmes A. Alleviating anxiety and taming trauma: Novel pharmacotherapeutics for anxiety disorders and posttraumatic stress disorder. Neuropharmacology. 2023;226: 109418. doi:10.1016/j.neuropharm.2023.109418

78. Palagini L, Miniati M, Caruso V, Alfi G, Geoffroy PA, Domschke K, et al. Insomnia, anxiety and related disorders: a systematic review on clinical and therapeutic perspective with potential mechanisms underlying their complex link. Neurosci Appl. 2024;3: 103936. doi:10.1016/j.nsa.2024.103936

79. Gonzalez-Diaz SN, Martin B, Villarreal-Gonzalez RV, Lira-Quezada CE de, Macouzet-Sanchez C, Macias-Weinmann A, et al. Psychological impact of the COVID-19 pandemic on patients with allergic diseases. World Allergy Organ J. 2021;14: 100510. doi:10.1016/j.waojou.2021.100510

80. Zhou Y, Liu A, Pu Z, Zhou M, Ding H, Zhou J. An investigation of the psychological stress of medical staff in Shanghai shelter hospital during COVID-19. Front Psychol. 2023;14: 1–7. doi:10.3389/fpsyg.2023.1083793

81. He C, Igwe N, Damian C, Feder A, Feingold J, Ripp J, et al. Racial & ethnic differences in mental health outcomes and risk factors among frontline healthcare workers during the COVID-19 pandemic. Gen Hosp Psychiatry. 2023;85: 1–7. doi:10.1016/j.genhosppsych.2023.09.003

82. Yardley P, McCall A, Savage A, Newton R. Effectiveness of a brief intervention aimed at increasing distress tolerance for individuals in crisis or at risk of self-harm. Australas Psychiatry. 2019;27: 565–568. doi:10.1177/1039856219848835
