# Supplementary material for: Impact of crisis intervention on mental health in the context of specific civilian emergencies
Source: PLoS One. 2025 Sep 10;20(9):e0331249. doi: 10.1371/journal.pone.0331249 (PMC12422494; doi:10.1371/journal.pone.0331249)
Supplement: Attachment — Submitted filename: Response to reviewers.docx [file pone.0331249.s004.docx]

Manuscript title: **Impact of crisis intervention on mental health in the context of emergencies: A scoping review**

Manuscript Ref: **PONE-D-24-11145**

Dear Honorable Editor and Reviewers,

We would like to show our sincere gratitude to the honorable editor and respected reviewers for giving us a chance to resubmit the manuscript. We believe that the revised version has fulfilled all the lacking of the manuscript and improved its quality. We have used blue color to show the correction marks throughout the manuscript.

**Reviewer #1’s comments**

**General comments**

1. Is the manuscript technically sound, and do the data support the conclusions?

Yes.

2. Has the statistical analysis been performed appropriately and rigorously?

Yes.

3. Have the authors made all data underlying the findings in their manuscript fully available?

No.

4. Is the manuscript presented in an intelligible fashion and written in standard English?

Yes.

Author’s reply: Many thanks to respected reviewer for kind observation. We have addressed all issues in the respective section point-by-point.

**Specific comments**

Reviewer: Ethics involves the application of fundamental ethical principles to research activities which include the design and implementation of research, society and others, the use of resources and research outputs, scientific misconduct and the regulation of research. Given the importance of ethics for the conduct of research, it should come as no surprise that many different professional associations, government agencies, and universities have adopted specific codes, rules, and policies relating to research ethics. Many government agencies have ethics rules for funded researchers.

*Author’s reply*: Thank you so much for the detailed review of the manuscript. We have followed the ethical guidelines throughout our research.

We are grateful to you for your kind suggestions, time and input for us. We belief your constructive suggestion helps us a lot. Without your kind observation, it would not be possible to improve the manuscript’s quality.

**Reviewer #2’s comments**

**General comments**

1. Is the manuscript technically sound, and do the data support the conclusions?

Yes.

2. Has the statistical analysis been performed appropriately and rigorously?

N/A.

3. Have the authors made all data underlying the findings in their manuscript fully available?

No.

4. Is the manuscript presented in an intelligible fashion and written in standard English?

Yes.

Author’s reply: Many thanks to respected reviewer for kind observation. We have addressed all issues in the respective section point-by-point.

**Specific comments**

Reviewer: Thank you to the authors for this very important and enriching scoping review. I suggest the following revisions before publication.

*Author’s reply*: Thank you so much for the detailed review of the manuscript. We have addressed all the suggestions point-by-point and responded to all comments below. The line numbers show the corrections of the revised manuscript.

Reviewer: 1. Introduction: The importance of this topic would be even better if you present some quantitative data, e.g. Charlson et al (2017) in Lancet found that 22% of people affected by conflict suffer from psychological disorders.

*Author’s reply*: Thank you so much for your suggestion. We have added some statistics in the first paragraph of the introduction section according to your kind suggestion.

Reviewer: 2. Methodology:

- Key words could have been broader (psychosocial interventions, psychosocial consequences, acute crisis, mental well-being, psychosocial impact, traumatic event..)

- can you explain why conflict settings have been excluded, as they are a humanitarian crisis? They are not mentioned anywhere

- Align the research questions/focus that you mention in the Abstract, Methodology, Research Protocol etc. They seem to be slightly different.

*Author’s reply*: We appreciate your critical observation.

-We have revised our keywords section of the research protocol to align with our search string.

-We have added a limitation regarding the exclusion of conflict settings in our conclusion section. Conflict settings have been excluded to maintain a specific focus on natural hazards and public health emergencies, ensuring a clear analysis of intervention strategies pertinent to these contexts. Future research could separately address conflict settings to provide a detailed understanding of crisis interventions in those contexts. Please see the conclusion section.

-We have revised our research question section of the research protocol to align the research question with abstract (overall research problem), introduction and methodology section.

Reviewer: 3. Results:

o Numeration is wrong

o Table 1: What do you mean with research start time given in months?; include the population

o Table 3: revise table, unclear what are the numbers behind anxiety e.g. Is pressure an official psychological term?

o 2.4 The relationship between crisis intervention and mental health: The reasoning of the interpretation of the spiderweb is difficult to follow. I suggest a quick presentation of the results before the interpretation; how have the themes been chosen? Also, there are some words, such as article, literature, participant that I do not see as key words and could be excluded.

o For the chapters 2.5.1 Direct impact and 2.5.2 indirect impact, there are only 2, respective 1 article that are being discussed that seems to be little.

*Author’s reply*: Many thanks for your critical observations and suggestions.

-We have corected numeration. Please see the results section.

- "Research Start Time" should be "Intervention Time". The "Intervention Time" given in months indicates the period during which the studies began their intervention. For example, "Intervention 0-Jan" refers to studies that started interventions immediately or within the first month. Please see the revised Table 2.

- We appreciate your critical observation. We have revised the mentioned table. “Pressure” should be ‘Stress”. We are sorry for our typos. Please see the revise Table 4.

- We have generated the Figure 2 again using VOS viewer, and added it. We have also updated the explanation.

Reviewer: 4. In the annexe, I would suggest one table with all the articles to quickly be able to jump to the articles with main features.

*Author’s reply*: Thanks for your kind suggestions. We have added the list of the selected documents as “Supplementary 2. Selected documents for in-depth analysis”.

Reviewer: 5. Be careful with terminology “this study provides evidence...” which is not the goal of a scoping review; or “direct effect on mental health...” that is only applicable if it was a RCT that controlled for other variables.

*Author’s reply*: We appreciate your critical observations, and suggestions. We have checked the whole manuscript and corrected the wording.

Reviewer: 6 Wishing you good continuation of your research, this is what is needed!.

*Author’s reply*: Many thanks for your suggestions. We have revised and updated the conclusion section by incorporating your all suggestions.

We are grateful to you for your kind suggestions, time and input for us. We belief your constructive suggestion helps us a lot. Without your kind observation, it would not be possible to improve the manuscript’s quality.

**Reviewer #3’s comments**

**General comments**

1. Is the manuscript technically sound, and do the data support the conclusions?

Yes.

2. Has the statistical analysis been performed appropriately and rigorously?

N/A.

3. Have the authors made all data underlying the findings in their manuscript fully available?

Yes.

4. Is the manuscript presented in an intelligible fashion and written in standard English?

No.

Author’s reply: Many thanks to respected reviewer for kind observation. We have addressed all issues in the respective section point-by-point.

**Specific comments**

Reviewer: 1. The manuscript is technically sound, the rationale, the methodology, presentation of results, discussions and conclusions are coherent. The methodology was rigorous, and conclusions were made based on results. All the research inquiries were answered by the results of the study.

*Author’s reply*: Thank you so much for the detailed review of the manuscript. We have addressed all the suggestions point-by-point and responded to all comments below. We have used blue color to mark the corrections. The line numbers show the corrections of the revised manuscript.

Reviewer: 1. The authors are recommended to include the keywords used for document selection and the major findings of the key research in the relevant section.

*Author’s reply*: We appreciate your kind observation and comment. We have added the keywords used for document selection in our research protocol Table. Please see Table 1. Besides, we have also added the keyword network analysis in the section 3.4 with Figure 2 under results section.

Reviewer: 2. Narrative synthesis was used to simply put the different findings together. It is suitable for scoping studies due to its adaptability in accommodating different study designs. Statistical analysis is not applicable for the study design.

*Author’s reply*: We appreciate your critical observation. In our study, we focused on social, economic and environmental vulnerability, and discussed in detail. Please see sub-section 4.2.2.

Reviewer: 3. Data can be made available upon request.

*Author’s reply*: Many thanks for suggestion. We have added the list of the selected documents as “Supplementary 2. Selected documents for in-depth analysis”.

Reviewer: 4. There manuscript has some clumsy sentences and some grammar errors to be corrected.

*Author’s reply*: Thanks for your kind suggestions. We have checked the whole manuscript and corrected the clumsy sentences and grammar errors. Please see the highlighted texts.

We are grateful to you for your kind suggestions, time and input for us. We belief your constructive suggestion helps us a lot. Without your kind observation, it would not be possible to improve the manuscript’s quality.
