# Supplementary material for: Impact of crisis intervention on mental health in the context of specific civilian emergencies
Source: PLoS One. 2025 Sep 10;20(9):e0331249. doi: 10.1371/journal.pone.0331249 (PMC12422494; doi:10.1371/journal.pone.0331249)
Supplement: Attachment — Submitted filename: Response_to_reviewers_auresp_2.docx [file pone.0331249.s005.docx]

Manuscript title: **Impact of crisis intervention on mental health in the context of emergencies: A scoping review**

Manuscript Ref: **PONE-D-24-11145**

Dear Honorable Editor and Reviewers,

We would like to show our sincere gratitude to the honorable editor and respected reviewers for giving us a chance to resubmit the manuscript. We believe that the revised version has fulfilled all the lacking of the manuscript and improved its quality. We have used blue color to show the correction marks throughout the manuscript.

**Journal Requirements:**

Please review your reference list to ensure that it is complete and correct. If you have cited papers that have been retracted, please include the rationale for doing so in the manuscript text, or remove these references and replace them with relevant current references. Any changes to the reference list should be mentioned in the rebuttal letter that accompanies your revised manuscript. If you need to cite a retracted article, indicate the article’s retracted status in the References list and also include a citation and full reference for the retraction notice.

Author’s reply: Thank you for your valuable feedback on our manuscript. We have carefully reviewed the reference list to ensure that it is complete, accurate, and up-to-date. Additionally, we did not cite any retracted papers in our manuscript.

**Reviewer #1’s comments**

1. If the authors have adequately addressed your comments raised in a previous round of review and you feel that this manuscript is now acceptable for publication, you may indicate that here to bypass the “Comments to the Author” section, enter your conflict of interest statement in the “Confidential to Editor” section, and submit your "Accept" recommendation.

No Response

2. Is the manuscript technically sound, and do the data support the conclusions?

Yes.

3. Has the statistical analysis been performed appropriately and rigorously?

Yes.

4. Have the authors made all data underlying the findings in their manuscript fully available?

No.

5. Is the manuscript presented in an intelligible fashion and written in standard English?

Yes.

6. Review Comments to the Author

Please use the space provided to explain your answers to the questions above. You may also include additional comments for the author, including concerns about dual publication, research ethics, or publication ethics. (Please upload your review as an attachment if it exceeds 20,000 characters)

Reviewer #1: No Response

Author’s reply: Many thanks to respected reviewer for kind observation. We have addressed all issues in the respective section point-by-point.

**Reviewer #2’s comments**

**General comments**

**Reviewer #1’s comments**

1. If the authors have adequately addressed your comments raised in a previous round of review and you feel that this manuscript is now acceptable for publication, you may indicate that here to bypass the “Comments to the Author” section, enter your conflict of interest statement in the “Confidential to Editor” section, and submit your "Accept" recommendation.

**Response: All comments have been addressed**

Author’s reply: Many thanks to respected reviewer for kind observation, and acceptance. We have addressed all issues in the respective section point-by-point.

2. Is the manuscript technically sound, and do the data support the conclusions?

Yes.

Author’s reply: We appreciate your kind observation.

3. Has the statistical analysis been performed appropriately and rigorously?

N/A.

Author’s reply: Many thanks.

4. Have the authors made all data underlying the findings in their manuscript fully available?

Yes.

Author’s reply: We appreciate your kind observation.

5. Is the manuscript presented in an intelligible fashion and written in standard English?

Yes.

Author’s reply: We appreciate your kind observation.

6. Review Comments to the Author

Please use the space provided to explain your answers to the questions above. You may also include additional comments for the author, including concerns about dual publication, research ethics, or publication ethics. (Please upload your review as an attachment if it exceeds 20,000 characters)

Reviewer #3: Some of the clumpsy sentences are clupmsy. There is an inconsistency in the use of terms, for instance in the introduction, the use of crisis treatments in the place of crisis intervention.

Author’s reply: Many thanks to respected reviewer for kind observation. We have checked the whole manuscript and corrected the clumpsy sentences. Please see the blue marked sentences of the whole manuscript.

**Reviewer #4’s comments**

**General comments**

1. If the authors have adequately addressed your comments raised in a previous round of review and you feel that this manuscript is now acceptable for publication, you may indicate that here to bypass the “Comments to the Author” section, enter your conflict of interest statement in the “Confidential to Editor” section, and submit your "Accept" recommendation.

No Response

2. Is the manuscript technically sound, and do the data support the conclusions?

Partly.

Author’s reply: We appreciate your kind observation. We have addressed your kind concern in the revised manuscript.

3. Has the statistical analysis been performed appropriately and rigorously?

N/A.

Author’s reply: Many thanks.

4. Have the authors made all data underlying the findings in their manuscript fully available?

Yes.

Author’s reply: We appreciate your kind observation.

5. Is the manuscript presented in an intelligible fashion and written in standard English?

Yes.

Author’s reply: We appreciate your kind observation.

6. Review Comments to the Author

Please use the space provided to explain your answers to the questions above. You may also include additional comments for the author, including concerns about dual publication, research ethics, or publication ethics. (Please upload your review as an attachment if it exceeds 20,000 characters)

Reviewer #4: This title of this paper is "Impact of crisis intervention on mental health in the context of emergencies". My strong recommendation is to change the title to say "**Impact of crisis intervention on mental health in the context of specific civilian emergencies**" The paper leaves out a huge literature, not only on conflict zones, but also on the impact of genocide, terrorist attacks, etc. About one third of the articles focus on COVID, and most of the other articles look at clinical emergencies and responses by law enforcement and health care workers. The introduction to the article should make this clear from the outset. To elaborate further, there is a huge literature on terrorist attacks (such as 9/11), genocides (such as in Rwanda), the atrocities of war (right now in Gaza, Ukraine and Sudan, to give a few examples) and the overlapping profound consequences of rape. The authors barely touch on this very vast literature. Therefore, throughout the paper, the authors need make their focus on specific civilian events clear and avoid references to the many topics that they barely cover.

Author’s reply: Many thanks to respected reviewer for kind observation. We have revised the title to "Impact of Crisis Intervention on Mental Health in the Context of Specific Civilian Emergencies" to clearly reflect the study’s focus on civilian emergencies not associated with armed conflict, such as natural disasters and public health crises. Additionally, we have incorporated a clarifying statement in the Introduction that acknowledges the extensive research on mental health impacts in conflict settings, including terrorist attacks, genocide, and war-related trauma, while specifying that our study focuses on non-conflict, civilian emergencies. Please see the revised title, and other revision in the second paragraph of the introduction (lines 51-66, and 182-184), methodology (lines 190-193, 281-283, and table 1), results (lines 287-291, 371-376, 412-415, 433-434), discussion (lines 453-460, 467-468, and 603-605), and conclusion (lines 611-612) sections.

We are grateful to you for your kind suggestions, time and input for us. We belief your constructive suggestion helps us a lot. Without your kind observation, it would not be possible to improve the manuscript’s quality.
