# Supplementary material for: Impact of crisis intervention on mental health in the context of specific civilian emergencies
Source: PLoS One. 2025 Sep 10;20(9):e0331249. doi: 10.1371/journal.pone.0331249 (PMC12422494; doi:10.1371/journal.pone.0331249)
Supplement: Attachment — Submitted filename: Response_to_reviewers_auresp_3.docx [file pone.0331249.s006.docx]

Manuscript title: **Impact of crisis intervention on mental health in the context of specific civilian emergencies**

Manuscript Ref: **PONE-D-24-11145R2**

Dear Honorable Editor and Reviewers,

We would like to express our sincere gratitude to the honorable editor and respected reviewers for the opportunity to resubmit our manuscript. We believe that the revised version has addressed all the identified gaps and significantly improved the overall quality. All corrections have been highlighted in blue throughout the manuscript for ease of review.

**Journal Requirements:**

Please review your reference list to ensure that it is complete and correct. If you have cited papers that have been retracted, please include the rationale for doing so in the manuscript text, or remove these references and replace them with relevant current references. Any changes to the reference list should be mentioned in the rebuttal letter that accompanies your revised manuscript. If you need to cite a retracted article, indicate the article’s retracted status in the References list and also include a citation and full reference for the retraction notice.

Author’s reply: Thank you for your valuable feedback on our manuscript. We have carefully reviewed the reference list to ensure that it is complete, accurate, and up-to-date. Additionally, we did not cite any retracted papers in our manuscript.

**Reviewer #3’s comments**

1. If the authors have adequately addressed your comments raised in a previous round of review and you feel that this manuscript is now acceptable for publication, you may indicate that here to bypass the “Comments to the Author” section, enter your conflict of interest statement in the “Confidential to Editor” section, and submit your "Accept" recommendation.

No Response

2. Is the manuscript technically sound, and do the data support the conclusions?

Yes.

3. Has the statistical analysis been performed appropriately and rigorously?

Yes.

4. Have the authors made all data underlying the findings in their manuscript fully available?

Yes.

5. Is the manuscript presented in an intelligible fashion and written in standard English?

Yes.

Author’s reply: Thank you to the respected reviewer for your kind observation. We have addressed all issues in the respective section point-by-point.

**Reviewer #3’s comments**

Reviewer: 1. Most of the comments from the first review were addressed by the author.

Author’s reply: Thank you very much for your kind appreciations.

Reviewer: 2. The manuscript is technically sound and the data supports the conclusions.

Author’s reply: We appreciate your kind observations.

Reviewer: 3. The extracted data was analysed and presneted appropriately

Author’s reply: We appreciate your kind observations.

Reviewer: 4. Minor corrections:

52: The use of it's (it is) is not proper for a formal document line a research paper.

53,54,55: The author should note that a public Health emergency could be caused by an armed conflict due to the massive displacement of populations. It is thus important not to use the term to refer only to emergencies of infectious origin like COVID-19. The war in Ukraine and Gaza have resulted in public health emergencies in both settings. Disease outbreak (epidemic) is a better term to use.

51-65 has repetitive information about the focus of the study on emergencies that are not related to armed conflict. It could be presented in a less repititive and concise manner.

68-79: The sentence is long, and clumpsy. Make it shorter and clearer or use a conjunction and a present continous tense

412: Grammar

455-456: I would suggest you parahrase "The analysis focuses on public health interventions within civilian contexts, juxtaposing them with a selection of non-public health-related actions".

498: Repitition of demographic

596, 605: Grammar, assessments, and studies

Author’s reply: Thank you for your constructive feedback. We have carefully revised the manuscript in accordance with your suggestions. This includes correcting grammatical issues, improving sentence clarity, refining the use of formal language, and reducing repetition. We have also clarified the distinction between public health emergencies and epidemics, ensuring accurate terminology and a more concise presentation of the study’s scope. We believe these revisions have strengthened the overall clarity and quality of the manuscript. Please see the mentioned paragraphs.

**Reviewer #5’s comments**

1. If the authors have adequately addressed your comments raised in a previous round of review and you feel that this manuscript is now acceptable for publication, you may indicate that here to bypass the “Comments to the Author” section, enter your conflict of interest statement in the “Confidential to Editor” section, and submit your "Accept" recommendation.

Response: All comments have been addressed

2. Is the manuscript technically sound, and do the data support the conclusions?

Yes.

Author’s reply: We appreciate your kind observation.

3. Has the statistical analysis been performed appropriately and rigorously?

N/A.

Author’s reply: Many thanks.

4. Have the authors made all data underlying the findings in their manuscript fully available?

Yes.

5. Is the manuscript presented in an intelligible fashion and written in standard English?

Yes.

Author’s reply: Thank you to the respected reviewer for your kind observation. We have addressed all issues in the respective section point-by-point.

**General comments**

Reviewer: The author has revised well on the suggested and commented points. The authors have done their task. Good Job.

Author’s reply: We appreciate your comments and acceptance.

We are truly grateful for your valuable suggestions, time, and thoughtful input. We believe your constructive feedback has been immensely helpful. Without your observations, improving the quality of the manuscript would not have been possible.
