# Supplementary material for: Impact of crisis intervention on mental health in the context of specific civilian emergencies
Source: PLoS One. 2025 Sep 10;20(9):e0331249. doi: 10.1371/journal.pone.0331249 (PMC12422494; doi:10.1371/journal.pone.0331249)
Supplement: Attachment — Submitted filename: Response_to_reviewers_auresp_4.docx [file pone.0331249.s007.docx]

Manuscript title: **Impact of crisis intervention on mental health in the context of specific civilian emergencies**

Manuscript Ref: **PONE-D-24-11145R3 (Fourth round)**

Dear Honorable Editor and Reviewers,

We would like to express our sincere gratitude to the honorable editor and respected reviewers for the opportunity to resubmit our manuscript. We believe that the revised version has addressed all the identified gaps and significantly improved the overall quality. All corrections have been highlighted in blue throughout the manuscript for ease of review.

**Journal Requirements:**

1. If the reviewer comments include a recommendation to cite specific previously published works, please review and evaluate these publications to determine whether they are relevant and should be cited. There is no requirement to cite these works unless the editor has indicated otherwise.

Author’s reply: Thank you very much for your kind suggestion. The reviewers did not ask to cite any articles.

2. Please review your reference list to ensure that it is complete and correct. If you have cited papers that have been retracted, please include the rationale for doing so in the manuscript text, or remove these references and replace them with relevant current references. Any changes to the reference list should be mentioned in the rebuttal letter that accompanies your revised manuscript. If you need to cite a retracted article, indicate the article’s retracted status in the References list and also include a citation and full reference for the retraction notice.

Author’s reply: Thank you for your valuable feedback on our manuscript. We have carefully reviewed the reference list to ensure that it is complete, accurate, and up-to-date. Additionally, we did not cite any retracted papers in our manuscript.

Additional Editor Comments:

Authors, please note that this will be the last round of revision requests. Please address the comments raised by Reviewer 6.

Author’s reply: Thank you very much for your kind suggestions. We have addressed all issues in the respective section point-by-point.

**Reviewer #6’s general comments**

1. If the authors have adequately addressed your comments raised in a previous round of review and you feel that this manuscript is now acceptable for publication, you may indicate that here to bypass the “Comments to the Author” section, enter your conflict of interest statement in the “Confidential to Editor” section, and submit your "Accept" recommendation.

Reviewer #6: All comments have been addressed

2. Is the manuscript technically sound, and do the data support the conclusions?

Reviewer #6: Yes.

3. Has the statistical analysis been performed appropriately and rigorously?

Reviewer #6: N/A.

4. Have the authors made all data underlying the findings in their manuscript fully available?

Reviewer #6: Yes.

5. Is the manuscript presented in an intelligible fashion and written in standard English?

Reviewer #6: Yes.

Author’s reply: Thank you to the respected reviewer for your kind observation. We have addressed all issues in the respective section point-by-point.

**Reviewer #6’s comments**

Comment 1. This is an important, comprehensive and timely review on crisis intervention. The paper has already undergone several rounds of reviews, achieving significant improvements. This is due to the efforts of both the authors and the reviewers!

Author’s reply: Thank you very much for your kind appreciations.

Comment 2. From that point on, I was asked to participate as a new reviewer. Naturally, new reviewers find new points of criticism that can be improved. These include platitudes such as "Psychological crisis intervention is widely considered one of the most effective approaches for managing mental health crises" (lines 93-94), which are not cited and contribute nothing in terms of content. Or replace “Table 2. Summary of basic characteristics of the literature” with included studies: in PICO format, for quality assessment, or similar.

Author’s reply: We appreciate the reviewer’s careful reading and constructive feedback. In line with your suggestion, the sentence “Psychological crisis intervention is widely considered one of the most effective approaches for managing mental health crises” (formerly in lines 93–94) has been removed from the manuscript to avoid unsubstantiated generalizations and to maintain a focus on evidence-based statements. We have ensured that similar generic statements are either deleted or supported with relevant citations.

Regarding the suggestion to replace Table 2 with a PICO-format summary, we acknowledge the value of such structuring. However, Table 2 in our manuscript serves to present the basic characteristics of the included literature in alignment with the PRISMA-ScR framework for scoping reviews, which emphasizes descriptive mapping rather than PICO extraction. Since our objective was to provide an overview of study settings, populations, intervention timelines, and designs, we have retained the existing format, which we believe remains consistent with scoping review reporting standards.

Comment 3. However, in my opinion, the standard of the work in its current form is already sufficient for publication. If there really needs to be any changes to the review, I would shorten the introduction by lines 154-164. The three questions mentioned (160-164) are either not addressed in the review or are only addressed in passing. The central questions (which are significantly different) appear in Chapter 2.2 and are then adequately addressed in the review.

Author’s reply: We appreciate your kind observations. We have removed the mentioned sentences and did sub-sequent changes of the relevant contents in the methodology section to address your kind concerns. Please see lines 163-164, and Table 1.

Comment 4. Even though I personally would have approached this review with different priorities, relevant content is presented here that will probably be frequently cited.

Author’s reply: We appreciate your observation. We are truly grateful for your valuable suggestions, time, and thoughtful input. We believe your constructive feedback has been immensely helpful. Without your observations, improving the quality of the manuscript would not have been possible.
